# Searching for the earliest archaeological record: insights from chimpanzee material landscapes

**DOI:** 10.1098/rsif.2024.0101

**Published:** 2024-08-21

**Authors:** Jonathan S. Reeves, Tomos Proffitt, Soiret Serge Pacome, Lydia V. Luncz

**Affiliations:** ^1^ Technological Primates Research Group, Max Planck Institute for Evolutionary Anthropology, Deutscher Platz 6, Leipzig, Germany; ^2^ Center for the Advanced Study of Human Paleobiology, The George Washington University, 800 22nd Street NW, Washington, DC, USA; ^3^ Interdisciplinary Center for Archaeology and the Evolution of Human Behaviour, Universidade do Algarve Campus de Gambelas, Faro 8005-139, Portugal; ^4^ Laboratoire de Zoologie et de Biologie Animale, Université Félix Houphouët-Boigny, Abidjan, 22 BP 582 Abidjan 22, Côte d’Ivoire, West Africa

**Keywords:** chimpanzees, stone tools, primate archaeology, nut cracking, landscape archaeology

## Abstract

The origin of tool use is a central question in human evolutionary studies. Plio-Pleistocene core and flake technologies represent the earliest evidence of tool use in the human lineage. Some suggest this form of tool use is probably pre-dated by a phase of percussive tool use. However, there is currently no evidence for such a record. The archaeological signature of solely percussive behaviours is not as well understood as that associated with cores and flakes. The durable nature of primate percussive stone tools and their by-products provide an opportunity to investigate what such a record looks like. Here, we present a landscape-scale study of the chimpanzee (*Pan troglodytes verus*) material culture from the Djouroutou Chimpanzee Project, Taï Forest, Cote d’Ivoire. This study explores the interplay between behavioural and environmental factors in shaping the stone record of nut cracking. Through a survey of nut-cracking sites, the available nut species, and raw materials, we show how resource availability influences the resulting material signature of nut cracking. These results also reveal the diversity of material signatures associated with a purely percussive material record. We gain insight into the range of signatures that may be associated with a pre-core and flake archaeological record, providing new expectations for an earlier record of tool use.

## 1. Introduction

The scale and frequency at which humans use tools are unprecedented in the animal kingdom [1]. As a result, understanding the origin of tool use remains a central question in human evolutionary studies [2]. Although currently debated, the earliest archaeological evidence of tool use documents the production and use of sharp cutting edges to process food resources at 3.3 Ma [3–7]. More conclusive evidence, dating to approximately 2.6 Ma (and possibly earlier) [8,9], documents the systematic production of sharp edges for various cutting tasks [10–14]. Comparative studies of a wide range of living primates have been used to suggest the origin of tool use extends beyond what is documented in the archaeological record [15–19]. Cladistic analysis and parsimony indicate that the last common ancestor of chimpanzees and humans was probably a tool user [16–18,20] (but see [21]). If this hypothesis is true, it would imply that the origin of tool use extends as far back as 5–8 Ma [17]. However, physical evidence supporting tool use before 3.4 Ma is limited.

The paucity of evidence, to some, is simply because most material traces do not preserve over such great time scales [17]. The vast majority of tools used by modern primates are produced from perishable materials such as wood, leaves and grasses [17,19], that only preserve under unique circumstances [22,23]. Nevertheless, five primate species use stones as tools for various behaviours, but primarily for percussive behaviours such as nut cracking [24–26], processing marine resources [27,28] and stone-on-stone percussion [29]. Although these percussive activities represent a small fraction of the broader primate tool repertoire, they leave behind an enduring material record [30]. The recovery of capuchin (*Sapajus libidinosus*) and chimpanzee (*Pan troglodytes verus*) percussive tools dating to thousands of years ago highlights the potential for the recovery of such activities in deep time [31,32].

Percussive tools are argued to also play a critical role in the evolution of hominin technology with some researchers contending that the *Pan–Homo* last common ancestor was a percussive tool user [18,33,34]. This highlights that percussive technology may have served as a precursor to core and flake technology [19,35]. Before the systematic production of sharp cutting tools, percussive tools would have been essential for accessing nutrient-rich marrow from carcass bones as well as other plant-based materials [35]. Moreover, the biomechanical actions associated with pounding activities could have been easily adapted to facilitate technical actions associated with intentional flake production [36]. Experimental and ethological studies have shown that the incidental detachment of flakes or angular fragments resulting from these percussive activities would have led to the accumulation of sharp-edged objects suitable for cutting tasks [18,37]. The production of such assemblages may have provided new sources of sharp cutting edges beyond naturally sharp-edged stones that some have argued have been used by hominins prior to 3.3 Ma [3]. However, testing this hypothesis depends on our ability to recognize a purely percussive archaeological record before the onset of core and flake technology.

Percussive materials such as hammerstones and anvils used for flake production have been readily identified in archaeological contexts [38,39]. These artefacts are frequently recovered in Plio-Pleistocene contexts based on signs of battering and localized regions of crushing on the surfaces of water-worn cobbles and tabular blocks [40–42]. Consequently, studies have extensively described the morphology and damage patterns associated with these percussive tools [43–45]. Most recently, methods that combine high-resolution three-dimensional modelling techniques with quantitative surface analyses have augmented our ability to describe damage patterns of percussive tools associated with behaviours [46,47]. Especially, the integration of these archaeological methods with primate material culture has provided valuable insights into the damage patterns associated with percussive implements [29,43,44,48,49]. For example, a study of macaque hammerstones demonstrated that distinct behaviours such as nut cracking, oyster foraging and snail shell processing could be distinguished based on the location and surface morphometry of the resulting damage traces [48]. Investigations of chimpanzee nut-cracking hammers and anvils illustrated how differences in raw material properties influence the observed damage traces [44].

While the characterization of percussive implements is essential, it is also important to situate them within their broader socio-ecological context, as these factors are known to influence percussive activities and their material expression [29,30,50–55]. For example, research conducted on western chimpanzees has revealed that the frequency of nut cracking depends on the proximity of suitable stones to fruiting nut trees [56–58]. This is because chimpanzees across long-standing field sites in West Africa are known to transport hammers and anvils short distances [19,45,59–62]. Nevertheless, the continuous reuse and transportation of nut-cracking implements over time increases the number of accessible nut trees, thereby increasing the frequency of percussive activities and providing learning opportunities for novice nut-crackers [51,55,58]. Western chimpanzees are known to crack open a variety of nut species that vary in their spatial distribution and mechanical properties [19,24,58,63]. As a result, the spatial distribution of different nut tree species and tool stones can structure the social context of percussive activities [58]. In the Taï forest, for instance, chimpanzees crack open *Panda oleosa* nuts, which are the hardest nuts currently known [61]. *Panda* nut trees are often widely dispersed and occur mostly on their own. In addition, the availability of stones large enough to be used as hammers to break through the nut’s hard shell is low, limiting the number of individuals that can participate in *Panda* nut cracking at any given time [45]. As a result, *Panda* nut cracking is often a solitary behaviour [52]. Conversely, *Coula edulis* nuts, one of the most available nuts in the Taϊ forest, are brittle and easy to crack open. In addition, *Coula* trees tend to be more clustered, allowing nut cracking to be a group activity [52]. However, it is important to mention that these conditions could be specific to the Taï forest.

Nevertheless, the interplay between behavioural, material and environmental factors, in turn, has been shown to influence their material expression at the landscape scale [58,59,64,65]. Generative simulations illustrate how the interaction between resources, tool reuse and transport affect the scale and patterning of the resulting material record [51]. For example, sites in environments where resources are dispersed form localized clusters at the location of the food resource. Whereas, environments rich in food resources can facilitate landscape-scale archaeological patterning [51,58]. At the long-term field site of Bossou, Guinea researchers found that areas subject to frequent nut-cracking events possessed a greater number of materials than those less frequently used [58]. Moreover, patterns of tool transport and continuous use can influence the representation of recognizable tools [59,64]. Analysis of the nut-cracking assemblage from Panda 100 found a predominance of angular stone debris, whereas functionally identifiable hammerstones were notably absent from the assemblage, despite having been observed at the site before its abandonment [30]. Thus, when raw material availability is low and discarded tools retain utility, the repeated transport and reuse of hammers may prevent them from entering the archaeological record in a recognizable form [30,64].

Furthermore, the mechanical properties that influence how often percussive implements fracture also influence the size and the density of the debris accumulated at a site [30,64,66]. Studies have also shown the hardness of nuts cracked varies and influences the mass of stones chimpanzees select for use as hammers and the likelihood of producing angular debris through breakage [60,67]. Thus, factors related to transport and breakage play a role in shaping the density and composition of nut-cracking assemblages and ultimately their visibility in the archaeological record [68].

As a result, understanding the intersection of percussive activities, tool transport, the availability and characteristics of stone materials and the mechanical properties of processed food materials is, therefore, crucial for understanding the diversity of material signatures. Such an understanding of the percussive record is essential if we aim to identify an archaeological record associated with a deeper emergence of stone tool use. Here, we apply landscape archaeological methods to the primate material culture, to shed new insights into the diversity associated with a purely percussive material record. To do so, we investigate how the combination of chimpanzee nut-cracking behaviours and environmental factors produce diversity in the archaeological record of wild chimpanzees from the field site of the Djouroutou Chimpanzee Project, Taï National Park (Cote d’Ivoire). We conducted a comprehensive survey documenting the density, composition and representation of raw materials at 52 nut-cracking assemblages across a raw material and food resource-diverse study area. By doing so, we characterize how behavioural processes such as stone selection and transport, as well as environmental factors such as material properties and abundance, nut species and tool-site location contribute to the formation of the chimpanzee archaeological record. This provided an opportunity to establish chimpanzee tool-using behaviour to its material signature at the landscape scale. Through this investigation, we gain insights into the formation processes associated with the chimpanzee archaeological record. Given the purely percussive nature of this record, the material patterns documented in this study provide a means by which to discuss how the earliest forms of tool use in the hominin lineage may manifest as a material record.

## 2. Study area

**Figure 1.**
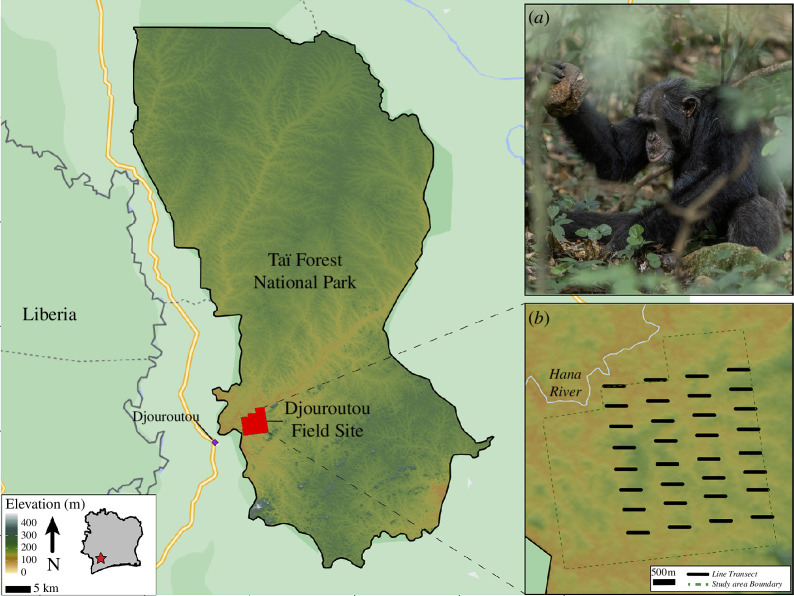
A map of the Taï Forest and the location of the study area. Insets: (*a*) An example of a chimpanzee cracking a nut using a stone hammer (Photo credit: Hans-Peter Schaub). (*b*) A map of the study area and the implementation of the line transect survey.

Situated 60 km to the southwest of the Taï Chimpanzee Project, Côte d’Ivoire (5°50′ N, 7°21′ W), the field site of the Djouroutou Chimpanzee Project (hereafter Djouroutou) is home to approximately 60 wild chimpanzees (figure 1). This group occupies a territory of about 25 km^2^ [69]. These chimpanzees are known to engage in regular nut cracking, using both wooden and stone tools composed of various raw materials [69]. The Djouroutou field site represents an ideal location for examining the influence of the environment on the material signature of nut cracking, given its landscape. The nut species and rock types present at Djouroutou vary in terms of their mechanical properties and representation across space, thus, providing an excellent opportunity to investigate how various ecological factors shape nut-cracking behaviours and their resulting material signature.

Observations have shown that the Djouroutou chimpanzees crack four species of nuts regularly: *Panda oleosa*, *Sacoglottis gabonensis*, *Parinari excelsa* and *Coula edulis*. These nut species exhibit vast differences in hardness, with *Panda* nuts being the hardest, requiring a force of 12.21 kN to break [61]. *Parinari* nuts are the second hardest, requiring a force value of 7.98 kN [61]. There is currently no hardness measurement for *Sacoglottis* nuts. However, studies examining the number of strikes required to crack various nut species show that *Sacoglottis* are softer than *Parinari* but harder than *Coula* nuts (see [60,70]). *Coula* nuts are the easiest to crack, with a force value of 2.72 kN [60].

**Figure 2.**
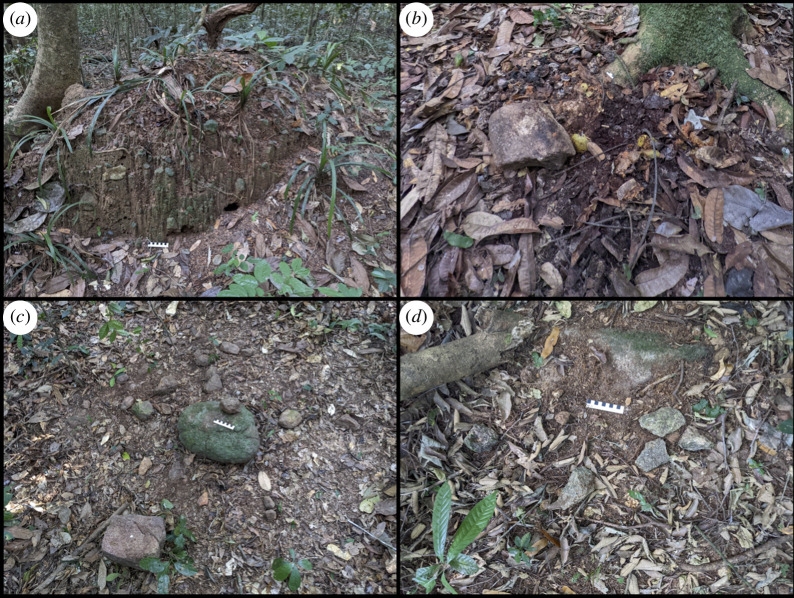
(*a*) An example of a fallen tree that has exposed quartz suitable for use as percussive implements. (*b*) An example of a single hammer discarded after use at a *Panda* nut tree. (*c*) An example of a *Coula* nut-cracking site with a stone anvil and quartz hammers. (*d*) An example of the mica-granite fragment associated with a small stone anvil.

The chimpanzees of Djouroutou crack nuts with wood as well as three recognizable rock types: quartz, metamorphosed granite (mica-granite) and granodiorite. These rock types were identified on the visual recognition of specific minerals diagnostic of the rock type and are in line with the broader geology of the Taï forest [71,72]. Line transects indicate that these rocks are distributed across the region in a northwest–southeast trajectory, aligning with the general topography of the study site [69]. The study site has undulating terrain, encompassing densely vegetated forest floors to steep inselbergs, which serve as the primary sources of mica-granite and diorite in the form of large cobbles and partially exposed boulders. Towards the northwest, the study site consists of low-lying areas dominated by swamp environments. Moving to the southeast, the elevation increases and the sediment becomes drier and gravelly, containing an abundance of quartz ranging from small pebbles to semi-exposed boulders. These semi-exposed boulders and inselberg exposures often serve as anvils for nut cracking. During the rainy season, active erosion is likely to increase the exposure of mica-granite and diorite and can move cobble-sized pieces of stone suitable for use as hammers to the base of their associated inselbergs. The undulating terrain funnels rainwater into an extensive network of stream channels, which incise the landscape and, in the process, carry and aggregate quantities of quartz on gravel bars in the low-lying areas. Quartz is also found within sediments across Djouroutou and can be accessed at places where sediment is exposed. In addition, fallen trees can act as sources as their root systems expose dirt and quartz to the surface when they are uprooted (figure 2).

The three available rock types in the study site exhibit variations in their material properties. On the Mohs hardness scale, which ranges from 1 (softest) to 10 (hardest) [73], all three rock types have relatively high hardness values, ranging between 5.5 and 8. While mica-granite is harder than quartz and diorite, chemical alteration processes to this material at Djouroutou have made it considerably softer compared with the other two rock types. Considering the observed variation in material properties and availability of stones and nuts within the study area, Djouroutou presents a unique opportunity to examine how environmental factors influence the variability in chimpanzee nut-cracking assemblages.

## 3. Data collection methods

To investigate the role of environmental factors on the formation of Djouroutou’s lithic assemblages, our study used a comprehensive landscape-scale dataset to address the following objectives: (i) assess the spatial distribution of rock types and nut tree species throughout the study area, (ii) describe the composition and character of stone nut-cracking assemblages within Djouroutou, and (iii) investigate the ecological factors (e.g. nut species distribution and rock type) influencing landscape-scale variation in nut-cracking assemblages. To characterize the spatial distribution of stone and nut trees, we conducted a survey using eight, 5 m wide line transects spanning the study area in a west–east direction. These transects were positioned at regular intervals of 500 m from each other running north to south, covering a total approximate distance of 5 km. To ensure coverage, each line was further divided into four equally spaced sub-lines, each measuring 500 m in length (figure 1).

Data were collected along each 500 m line segment using sampling plots at 20 m intervals. At each sampling plot, we recorded the presence of stone suitable for tool use, and the species of nut-bearing trees. Whenever we encountered a nut tree on these line transects we further investigated a 20 m radius (the nut falling zone) from the stem of the tree. Within this area, we recorded nut-cracking sites and the availability of tools and rocks. The stones deemed suitable for nut cracking were further characterized in terms of their mass, rock type and geometric properties. To not disrupt the natural foraging behaviour of chimpanzees, these data were collected in the absence of any chimpanzees in the area. In addition, to enhance the accuracy of our findings, we augmented the spatial distribution data of nut trees and nut-cracking sites by recording previously unidentified locations during focal follows designed to collect behavioural data throughout the year. The real-world coordinates for each tree were recorded as northings and eastings (UTM zone 29 N, WGS 1984).

Since nut cracking is a percussive activity, the hardness of the rock types and how often they break are likely to influence the amount of debris that accumulates at a nut-cracking site and ultimately what enters the material record. To assess the material properties of the rock types present in the Djouroutou area, we used a Proceq digital Schmidt hammer to measure rebound hardness following Braun *et al*. [74]. This approach enables the quantification of a rock’s resistance to strain, which directly relates to its propensity to break. In total, we collected hardness data for 100 individual rock samples of each material type. For each sample, we calculated the average hardness value based on 10 hardness measurements. This averaging technique is standard practice when collecting hardness measurements, as it minimizes the potential influence of small localized inconsistencies in the rock that could act on any single measurement [75].

### 3.1. Sampling stone nut-cracking sites

To generate a sample of nut-cracking assemblages across Djouroutou, we used a stratified random sampling strategy [76]. A stratified random sampling procedure provides a way to randomly select the assemblages to be sampled while also ensuring comprehensive coverage of the study area. Similar methods have been successfully applied to document spatial variation in material culture among long-tailed macaques [66]. To implement this procedure the study area was divided into a grid comprising 100 × 100 m squares. Within each square, we randomly selected previously mapped nut-cracking sites using a random number generator. Nut-cracking sites were defined as locations that possessed battered elements (i.e. stone with evidence of being used as hammerstones), an anvil and broken nutshells [77]. The location of each selected site was recorded using a Garmin inReach SE+ GPS. For each location, we recorded the primary nut species cracked based on the species of the nutshells surrounding the anvil. Stone tools and debris were recorded within a 10 × 10 m area surrounding the nut-cracking site, with the anvil serving as its centre. This sampling area was chosen to ensure that the stone assemblage associated with the target anvil was sufficiently sampled.

Each object recorded within the assemblage was characterized using standard analytical techniques in primate archaeology [30,50]. All stone tools and their by-products were identified based on clear traces of percussive activity (i.e. freshly fractured surfaces, traces of battering and crushing, and spatial association with broken nutshells). Each object was characterized—following Proffitt *et al*. [50]—in terms of its material type (i.e. diorite, mica-granite, quartz and wood) and assigned a specific artefact type, including complete hammerstone, broken hammerstone, detached pieces such as angular debris and anvil. A comprehensive description of each artefact type is provided as supplementary material (electronic supplementary material, ESM1). Length, width, thickness and mass were also recorded for each artefact. In instances where multiple anvils were found within a given sampling square, we also recorded the anvil that each recorded piece was closest to as well as its distance from the nearest anvil.

## 4. Ethical considerations

Chimpanzees are followed frequently to collect behavioural data throughout the year. In compliance with the health guidelines for the Djouroutou research site all observers wear surgical masks and maintain a minimum distance of 10 m from the chimpanzees at all times. In addition, no more than two observers were permitted to follow the same chimpanzee at any given time. To minimize the impact of our research on active nut-cracking sites, all materials included in this study were analysed in the field and left at the location where they were found. During the analysis of the materials, we also established a sanitation protocol to minimize the possibility of any cross-contamination when handling the stone material. To prevent the transmission of any pathogens from the analyst to the stone material via the air, we wore surgical masks whenever we were within 10 m of or incidentally encountered a nut-cracking site. Before analysis, each analyst would sanitize their hands with sanitizer and put on surgical gloves. Surgical gloves were always worn during the handling of stone materials. Upon completion of the analysis, the analysts would vacate the general vicinity of the nut-cracking site, remove their surgical gloves and sanitize their hands again.

## 5. Methods

### 5.1. Characterizing spatial distribution of nut trees and stone material

The spatial distribution of nut trees and rock types was characterized using standard deviational ellipses (SDE) following Bivand *et al*. [78]. SDE is a common method for visualizing the overall patterns of point distribution within a specific area of interest [79]. The ellipse is generated by calculating the standard deviation and the mean centre of the *x* and *y* coordinates of the points of interest to define its axes. The resulting ellipse provides a way to identify if the distribution of points is elongated or has a preferred orientation. By applying SDE to each rock type and nut species, it is possible to determine whether they exhibit distinct spatial patterns within the study area.

### 5.2. Summary of nut-cracking assemblages

The lithic assemblages recorded in each 10 × 10 m sampling unit around anvils were summarized using a series of assemblage-level attributes. These attributes were chosen as they have been previously shown to vary across space [30,45,51,54,58,66]. Those included in the study were the number of anvils, number of hammers and number of fragments present. Comprehensive definitions of these attributes are provided as supplementary material (electronic supplementary material, ESM1). The total number of stone materials present was also calculated by summing the number of hammers and fragments present. Naturally occurring and immobile formations such as roots and exposed stone outcrops that were often used as anvils, were considered separate from the material modified by nut cracking and were excluded from this calculation, as their presence is a result of geological processes as opposed to the nut-cracking activity itself. The frequency and proportion of diorite, mica-granite and quartz were also calculated for each assemblage. Each assemblage was assigned a primary raw material type, which corresponds to the rock type comprising more than 50% of the assemblage. Furthermore, the minimum, mean and maximum weights of hammers and fragments were also determined. The presence of stone anvils was recorded as they have an important role in assemblage formation as they are harder than wooden anvils and probably cause stone hammers to break more frequently when compared with wooden anvils. To examine the effect of raw material availability on assemblage composition, the distance to the nearest mica-granite, diorite and quartz source was calculated based on the locations of the naturally occurring rocks [45].

### 5.3. Statistical comparisons

A series of statistical tests were conducted to examine the effects of environmental factors on the patterning observed within the nut-cracking assemblages. First, we used a Kruskal–Wallis test to determine whether the different rock types had different hardness values. A Kruskal–Wallis test was chosen over an analysis of variation because hardness values were non-normally distributed (Shapiro–Wilks: *W* = 0.94, *p* = 2.937 × 10^−6^). A Dunn’s test was used as a post hoc test to determine the significance of pair-wise differences between the rock types [80]. To test for the influence of nut species and raw material properties of stone present on nut-cracking assemblage composition we used a series of generalized linear models (GLMs) to examine the effects of the presence of stone anvil, nut species cracked and the primary raw material represented on the number of stones present, the number of hammers present and the size of stones present within an assemblage [81].

The significance of each model tested was determined using a likelihood ratio test to its difference from a null model consisting only of an intercept [82]. The significance of each predictor variable was determined by comparing the full model with reduced models where one of the predictor variables had to be removed using a likelihood ratio test [83]. All analyses were developed and implemented using R (v. 4.2.2) [84]. The significance of each model comparison was determined using an α-value of 0.05. During analysis, we checked various diagnostics of model validity and stability using variance inflation factors, leverage, measures of influence (DFBETA) and overdispersion.

#### 5.3.1. Density model

To investigate factors influencing artefact density, we used a GLM with a negative binomial error structure and a loglink function. The number of artefacts was specified as the response variable, with primary raw material present, nut species cracked and the presence of a stone anvil listed as predictor variables. A negative binomial error structure was chosen, as the number of artefacts comprises counts bounded by 0 on the lower end and infinity on the upper end [80]. Although a Poisson error structure is more commonly used for such data, a negative binomial model is preferred in this particular case as it deals with issues of over-dispersion present within the data [81].

#### 5.3.2. Hammer frequency model

The influence of the three predictor variables on the presence of hammers within an assemblage was also investigated using a model with the same structure as the aforementioned model. In this case, however, the number of hammers present was listed as the response variable.

#### 5.3.3. Stone mass model

We also tested the influence of nut species, primary material and presence of a stone anvil variables on the maximum mass of materials recovered at nut-cracking sites, given chimpanzees are known to choose hammers of different masses to crack nuts of varying hardness. To do so, we use a GLM with a Gaussian error structure. A Gaussian error structure, in this case, is preferred because, unlike counts of artefacts, the mass is free to vary continuously. The maximum mass of the material present was set as the response variable. Maximum mass was chosen over the mean mass because it is already known from previous studies that the process of nut-cracking produces a large number of small fragments that are disproportionate to the number of hammers present [30]. As a result, calculating the mean size would misrepresent the presence of larger pieces potentially diagnostic of nut-cracking activities associated with a different species. To capture these large pieces within the assemblage we choose to use the maximum size.

## 6. Results

### 6.1. Spatial distribution of nut trees and raw materials

**Figure 3.**
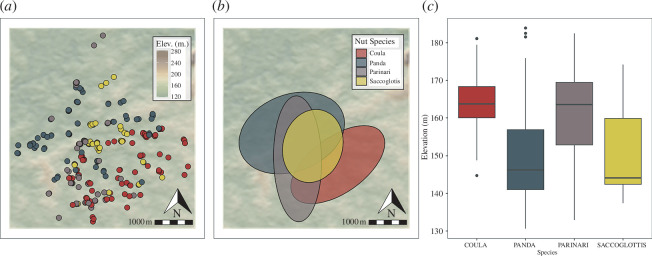
Plots showing the spatial distribution and topographic context of different nut tree species across Djouroutou. (*a*) The locations of individual nut trees according to their species. (*b*) Standard deviational ellipses showing general trends in the distribution of each nut tree species. (*c*) Boxplots visualizing the elevation associated with each tree species.

The survey of Djouroutou documented the locations of 289 nut-bearing trees. Results show distinct patterning in the spatial distribution of various nut tree species across the study area. *Panda* trees are generally concentrated in the northwest region and are largely associated with the low-lying areas in this section of forest (figure 3*a,b*). *Coula* trees are found in more elevated regions in the southeast. *Parinari* and *Saccoglottis* trees do not seem to be influenced by elevation and are thus more centrally situated within the study area (figure 3*c*). Specifically, *Parinari* trees generally occupy a narrow area of forest that extends from the northern portion to the southern extent of the study area. *Saccoglottis* trees are predominantly clustered in the central portion.

**Figure 4.**
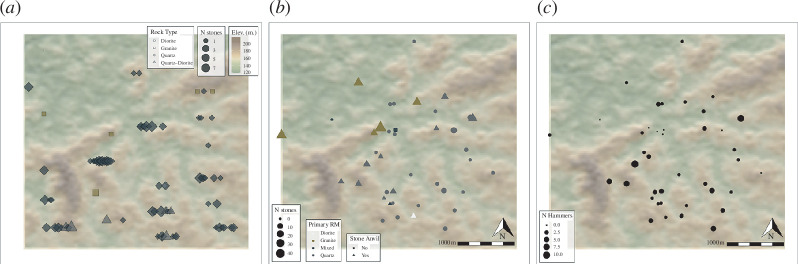
Maps of the study area illustrating the spatial patterning of available rocks and nut-cracking assemblages in Djouroutou. (*a*) A map visualizing the distribution of naturally available rocks according to their raw material and abundance. (*b*) A map showing the spatial distribution of nut-cracking sites, their density, the primary raw material represented and the presence of stone anvils. (*c*) A map showing the spatial distribution in the representation of hammers within sampled nut-cracking assemblages.

In terms of the availability of stone, a survey along the transect lines documented 223 rocks suitable for use as hammers from 86 different locations across the study area. These data show that the frequency and distribution of these raw material types are variable across space (figure 4). Quartz is most abundantly available, appearing in a total of 12% of areas sampled along the transects. Diorite was documented in slightly more than 1% of transect plots, and mica-granite appeared in less than 1% of the sampled plots. Only 1% of the sample plots contain more than one type of rock. Of this 1%, only quartz and diorite or quartz and mica-granite are represented in the same sample plot. There are no plots where both diorite and mica-granite are found.

A general lack of admixture between the three raw materials is owing to how they are distributed across the space. Quartz is the most widespread and overlaps with the locations of all four nut tree species. Nevertheless, it occurs most abundantly in the southern half of the study area where *Coula*, *Saccoglottis* and *Parinari* trees are found. Although there are a few localized instances of quartz in the northern third of the region, it is largely absent from this section of the study area. As a result, quartz is largely absent from the section of the forest where *Panda* trees are most frequent. In comparison with quartz, the spatial distributions of diorite and mica-granite are much more localized. Diorite overlaps considerably with the spatial distribution of *Parinari* and *Saccoglottis* as it occurs almost exclusively in the central portion of the southern two-thirds of the study area. Although *Coula* trees are situated in the southern third of the forest, there is some overlap with the northern third. Mica-granite occurs predominantly in localized quantities in the northern third of the study area and thus overlaps predominantly with the spatial distribution of *Panda* trees. There is, however, a single isolated location where mica-granite is present in the central third of the study area, but it does not overlap with natural occurrences of quartz and/or diorite.

**Figure 5.**
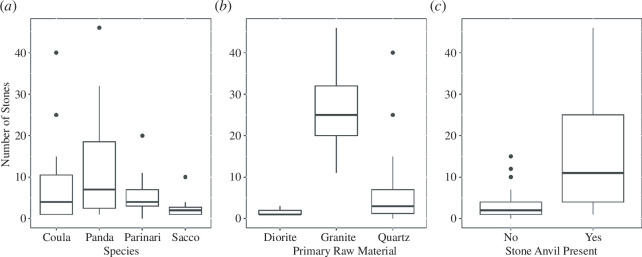
Boxplots showing the individual influences of the nut-species cracked (*a*), the primary raw material present (*b*) and the presence of a stone anvil (*c*) on the number of stones present within sampled nut-cracking assemblages.

In terms of their material properties, an analysis of the Schmidt hammer values reveals that there are significant differences in the hardness of the three rock types (Kruskal–Wallis, *X* ^2^: 96.201, d.f. = 2, *p* = 2.2 × 10^−16^). This result is driven by the fact that mica-granite is considerably softer than both quartz and diorite (Dunn’s test, electronic supplementary material, ESM2; figure 5). Mica-granite has an average hardness value of 19.3 as opposed to quartz and diorite which have hardness values of 47 and 49, respectively. While quartz exhibits a greater interquartile range in hardness values than diorite, these differences are not significant (Dunn’s test; electronic supplementary material, ESM2; figure 5).

### 6.2. Spatial variation in nut-cracking assemblages

The stratified random sampling procedure documented stone assemblages from 52 nut-cracking localities across Djouroutou. The composition of sampled assemblages shows that nut-cracking leaves a widespread and variable material signature across Djouroutou in terms of the representation of raw material types and artefact types. On average, each sampling square contains 14 stone artefacts. However, the number of artefacts per sampled unit ranges from a minimum of two to a maximum of 54. In terms of size, the maximum weight of the artefacts recovered in a single assemblage also varies considerably, ranging from fragments as small as 9 g to hammers as heavy as 9.5 kg. The frequency of hammerstones within any given sampling unit is also variable and ranges from 2 to 20, with some assemblages devoid of hammers. Likewise, the number of fragments present owing to hammer and/or anvil breakage ranges from 0 to 46.

In terms of the representation of different raw material types, most sites (83%) comprise a single raw material. Quartz is most frequently represented in nut-cracking assemblages, with 70% of the assemblages consisting solely of this raw material. Sites that consist primarily of mica-granite represent 11% of the landscape sample. There are only two sites that comprise entirely diorite (4.5%). Consistent with the pattern of toolstone availability, sites where more than one material type is present contain either quartz and diorite or quartz and mica-granite but never mica-granite and diorite. In addition, assemblages that contain more than one rock type consist of a primary material type and a secondary rock type that represents less than 10% of the overall assemblage. There are only two sites where the proportion of additional raw material is more equally represented, but the total number of artefacts recovered from these locations is less than five.

In general, the variation in the representation of material reflects the natural availability of stone across Djouroutou. Assemblages that comprise primarily mica-granite are situated in the northern third of Djouroutou where mica-granite is most frequently found (figure 3). In sites where mica-granite is present, the proportion of granite within an assemblage increases as the distance to locations of naturally occurring granite lessens. Similarly, assemblages containing diorite occur in proximity to where the stone naturally occurs. Sites containing primarily quartz lithics are as widespread as naturally occurring quartz (figure 3). However, there are few instances where quartz is documented beyond where it naturally occurs. There are three assemblages situated in the northern third of Djouroutou, predominately composed of mica-granite, that contain a percentage of quartz even though quartz is not naturally available.

Variations in the presence of stone anvils, density, number of hammers and the maximum size of artefacts also tend to follow a northwest-to-southeast trend. All but two of the sites that contain stone anvils are predominantly situated in the northern and central thirds of Djouroutou. Concerning artefact density, the total number of artefacts recovered from a sampling unit is greatest in the northwest and generally decreases towards the southeast. Interestingly, even though the sites with the greatest amount of material are situated in the northern third of the study area, the number of hammers documented at these sites is comparatively few. Hammers occur in greater frequencies in the central and southeastern third of the study area. Similarly, the maximum size of stones documented at nut-cracking sites is also greater in the central and particularly southern thirds of the study area.

### 6.3. Environmental influences on assemblage variation

The results of the GLMs reveal that much of the assemblage variability is associated with the nut species cracked, the presence of a stone anvil and the primary raw material represented at the site. The diagnostic analysis of the models found no issues with collinearity. However, the diagnostic analysis revealed three influential sites that impacted the stability of each model. Further inspection revealed that these sites all were unique in the very low number of artefacts documented at these influential sites (*n* < 2). All of these influential sites possess diorite as either the hammer or anvil present. The durability of diorite as a material may explain the low frequencies of materials at these sites. While removing them improved model stability, their exclusion did not change the overall effects and patterns revealed by each model. Therefore, these sites were retained in the analysis given their inconsequential nature. All code and detailed results associated with this analysis are provided as supplementary material.

#### 6.3.1. Density model

**Figure 6.**
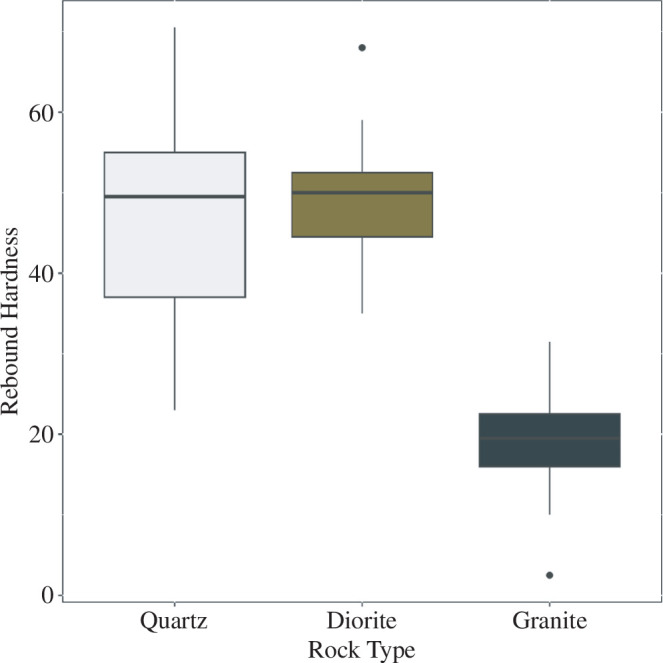
Boxplots comparing the hardness values of the different raw material types used for nut cracking in Djouroutou.

The results of the density model found that environmental factors have an influence on the quantity of stone material found at the nut-cracking site. The full model was found to be highly significant when compared with the null model (*X* ^2^: 41.029, *p* = 2.9 × 10^−7^). Full model–reduced model comparisons also determined that nut species, primary raw material and the presence of a stone anvil within the sampling area are highly significant. For nut species, both *Coula* (*E*_intercept_: −0.06) and *Panda* (*E*_SpeciesPanda:_ −0.49) nuts possess the greatest the number of stones within an assemblage, while *Saccoglottis* (*E*_SpeciesSacco_: −0.87) and *Parinari* (*E*_SpeciesParinari_:-.72) had fewer (*X* ^2^: 53.748, *p* = 0.045, figure 4*a*). Moreover, the primary raw material also has a significant effect on the number of artefacts (*X* ^2^: 65.568, *p* = 4.79 × 10^−5^). Assemblages that are primarily composed of granite (*E*_granite_: 3.0) had the greatest influence on the number of artefacts. Moreover, assemblages where the primary raw material is quartz had fewer artefacts than granite assemblages (*E*_quartz_: 1.82) but more than those compsed of diorite (figure 6). In addition, the presence of a stone anvil within the sampling square also has a positive effect on the number of artefacts documented (*E*_AnvilPresent_: 0.91, *X* ^2^: 55.709, *p* = 0.002). As such, sites, where a stone anvil is present, have significantly more artefacts present than in sampled areas where only wood anvils are present.

#### 6.3.2. Hammer frequency model

**Figure 7.**
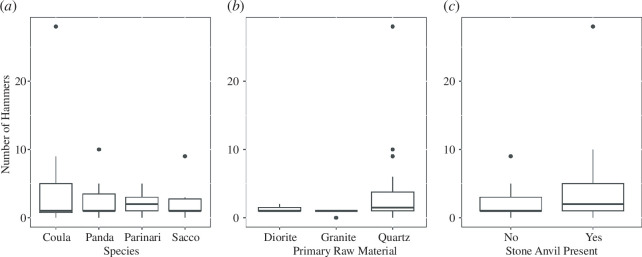
Boxplots showing the individual influences of the nut species cracked (*a*), the primary raw material present (*b*) and the presence of a stone anvil (*c*) on the number of hammers present within sampled nut-cracking assemblages.

Primary raw material present and the presence of a stone anvil were shown to influence the frequency of hammers present within nut-cracking assemblages (figure 5). A full-null model comparison determined that the hammer frequency model was highly significant compared with the reduced model (*X* ^2^: 20.10413, *p*: 0.003). Full model-reduced model comparisons revealed that the primary stone material present within the assemblage had a significant effect on the frequency of hammers within an assemblage (*X* ^2^: 13.86, *p*: 0.0001). Sites where the primary raw material is quartz (*E*_quartz:_ 1.3) have a greater representation of hammers than sites that are predominantly diorite (but more than those composed of diorite; figure 7). Sites that are predominantly composed of granite have the smallest representation of hammers (*E*_granite_: −0.92). The presence of stone anvils also has a significant positive effect on the number of hammers found at a site (*X* ^2^: 14.69, *p*: 0.0001; figure 7). This result indicates that there is a higher frequency of hammers in areas where stone anvils are present (*E*_anvilpresent_: 1.38). Nut species, however, did not have a significant effect on the number of hammers present (*X* ^2^: 0.98, *p*: 0.8).

#### 6.3.3. Assemblage mass model

**Figure 8.**
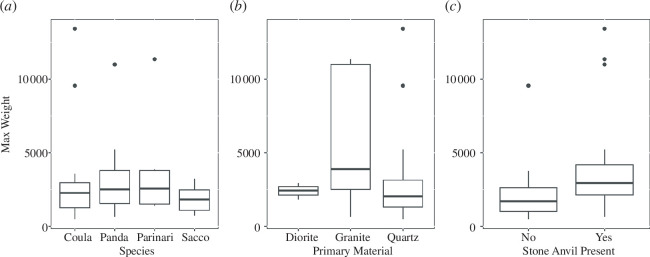
Boxplots showing the individual influences of the nut species cracked (*a*), the primary raw material present (*b*) and the presence of a stone anvil (*c*) on the size of stones present within sampled nut-cracking assemblages.

Nut species, primary raw material and stone anvil present did not influence the maximum size of stones (figure 8). The maximum size of artefacts at sites where the primary raw material is granite is, on average, heavier than those where diorite and quartz are dominant. In addition, the maximum mass of stone materials left at sites with stone anvils is greater than those with wood anvils (figure 8). However, the full-null model comparison determined that the influence of these variables on the maximum mass of the stones recovered from the nut-cracking sites was not significantly different from the null model (*X* ^2^: 81465146, *p*: 0.094). This result is counterintuitive given that it is known that chimpanzees select stones according to the mechanical properties of the nuts that they are cracking [60].

## 7. Discussion

### 7.1. The formation of chimpanzee assemblages at Djouroutou

Our results show that chimpanzee nut cracking at Djouroutou results in the creation of a structured and diverse material landscape. The observed material pattern at Djouroutou is influenced by spatially variable access to different species of nut trees and tool materials with distinct properties. This pattern allows us to discuss the ecological influences on the formation of the chimpanzee percussive record. Our data show that the different nut tree species occupy spatially distinct areas, probably influenced by the specific habitat requirements of each species. For instance, *Panda* nut trees, known to prefer seasonally flooded forest environments [85], predominantly occupy the northern third of the study area. On the other hand, *Coula* trees, whose root systems require permeable soil for effective drainage [86], are concentrated in the gravelly and more highly elevated southern region, whereas *Saccoglottis* and *Parinari* trees are generally focused in the central portions of the study area. In terms of raw material availability, the softer mica-granite is largely limited to the northern half of the region and the harder diorite is found in localized points in the southern half of the region where their associated inselbergs naturally occur. In contrast, quartz is more widely distributed since it can be found in the sediment throughout the study site. The varied distribution present across Djouroutou also implies that the physical attributes of available resources such as their hardness also vary across space.

Our results show that hardness plays a critical role in the formation of percussive assemblages because it has a direct influence on the likelihood that percussive implements will break during use. This notion is emphasized by the significant effect that the variables nut species, primary raw material and the presence of stone anvils have on the number of artefacts. In terms of primary material, softer stones, like mica-granite, require less force to damage [44] and will break more frequently than harder rocks such as quartz or diorite. The presence of stone anvils further promotes breakage, as stone-on-stone collisions during accidental mishits will cause hammers to accrue damage and break at a faster rate than when mishits involve softer wooden anvils. The hardness of nut species has a similar effect, as harder nuts such as *Panda* nuts require greater force to crack than *Coula* nuts*,* further promoting hammerstone breakage. In sum, factors that increase the likelihood of breakage will result in the greater production of fragments increasing the amount of material that accumulates at a given site.

Beyond the likelihood of breakage, nut hardness is also known to influence hammerstone selection as chimpanzees will often use larger hammerstones to achieve the force needed to crack harder nuts [60]. This pattern of selection will also influence the number of artefacts that accumulate at a given site, as larger hammers have more volume that can be broken down into smaller pieces, increasing the representation of fragments. On the other hand, softer nuts only require small hammers with less volume of stone that will break less often, contributing to the accumulation of few pieces. The observed differences in the density of artefacts across Djouroutou can be explained by the intersection of particularly hard *Panda* nuts, stone anvils and soft stone (mica-granite) in the north and the softer shelled nuts, wooden anvils and hard materials in the south.

The influence of hardness on breakage frequency also explains the relatively low occurrence of hammers in the northern third of the study site. The increased rate at which the soft mica-granite breaks apart implies that hammerstones may be quickly broken down into fragments, leading to an under-representation of identifiable hammers. Variation in availability and abundance of the material may also contribute to this pattern as studies have shown that chimpanzees will continuously re-use and move hammers as needed [45,59,60,62]. Thus, the general lack of available stone in the northern third of the Djouroutou study area may increase the incentive to transport and repeatedly use the few hammerstones that are available until exhaustion, further contributing to the lower representation of hammers present within nut-cracking assemblages in the region.

Concerning central and southern regions of the study area, the softer shells of the *Coula* and *Sacoglotis,* in combination with the availability of harder materials such as quartz and diorite, implies that hammers will remain in a more intact state, thus decreasing the number of artefacts present but increasing the representation of hammers within a given assemblage. Contributing to this general pattern, the widespread availability of quartz indicates that there is ample stone material in these regions. As a result, there is little need to continuously transport and re-use individual hammers owing to the high availability of quartz material (figure 3). Individual hammers are probably subjected to fewer uses and less likely to break, leading to greater representation of hammers in nut-cracking assemblages.

Interestingly, the intersection between use and material properties creates a lack of correlation between maximum artefact size and species of nuts being cracked, contradicting ethological observations that show that chimpanzees select the mass of the hammer according to the hardness of the nut [24,60,87]. The discrepancy between observations and record, however, is not unique to the Djouroutou study site [69]. In general, recorded *Panda* hammers, are almost always over 2 kg in mass [45], yet the assemblage associated with Panda 100 did not contain artefacts greater than 1 kg [30]. This pattern is probably a product of continuous hammer reuse described above. In addition to influencing the number of hammers and fragments present, the continuous breakage of percussive implements through the systematic reuse and transport of soft stone reduces the size of pieces that accumulate at nut-cracking sites. As a result, despite the larger size of hammers required, *Panda* cracking sites end up appearing more similar in artefact size to *Coula* cracking sites owing to the systematic breakage of hammers in the north of the study area.

### 7.2. Implications for chimpanzee behaviour

The abundance of fragments often present at sites attests to how repeated use of hammers ultimately causes them to break, creating a material record of past behaviours. Although the continuous use of hammers ultimately drives this pattern, the variation observed in nut-cracking assemblages from Djouroutou may also offer insights into chimpanzee tool transport. Modern observations of chimpanzees in the Taï forest have shown that stone hammers are continuously moved in and out of different nut-cracking sites as they are needed [24,45,61].

Therefore, the general paucity of hammers, particularly in the northern region of the study area, and the presence of fragments composed of specific raw materials suggest a dynamic pattern of movement of hammers across the landscape [24,60,60,62]. The isolated occurrences of quartz artefacts in the northwestern region of the study area where mica-granite locally occurs are of particular interest in this respect because there are not enough fragments to compose a single broken-down hammerstone. Thus, these isolated incidences further attest to the movement of relatively complete quartz hammers in and out of nut-cracking sites.

The presence of quartz pieces in regions of the forest where they do not naturally occur has implications for the influence of chimpanzee nut cracking on the broader distribution of stone material within the study area. Generally, chimpanzees are not known to transport tool materials over long distances in individual bouts [61]. However, they can displace tools over kilometres through continuous transport and reuse [45,51]. The presence of quartz in this area indicates that this raw material can be transported beyond its natural distribution [45,51]. This transport pattern seems to be unique to quartz, which may suggest preferential transport of this raw material over diorite and mica-granite. However, there is abundant evidence of the use of all three materials wherever they are locally available. Computational research has shown that tool transport distances are tied to how quickly a tool will become exhausted during repeated use [68]. Therefore, the hardness of quartz causes it to break less frequently than the softer mica-granite, allowing it to be moved at greater distances. Yet, diorite is of a similar hardness to quartz but remains locally constrained. This may indicate a degree of preference for quartz over diorite. However, the properties driving this preference are unknown.

### 7.3. Implications for hominin archaeology

Given that we cannot observe the past, generating expectations for how specific behaviours manifest as material patterns can be challenging. While modern analogues serve as a cornerstone for connecting behaviour to archaeological patterns, there is very little opportunity to observe stone tool use and discard within a living system. The stone tool-using behaviours of chimpanzees provide a rare opportunity to study the formation of a material record within its natural socio-ecological context. The capacity to do so is critical for the detection and interpretation of archaeological material, as this work allows us to explicitly establish connections between behaviour, the broader environment and material patterning. The correlation between assemblage composition and the mechanical properties and spatial distribution of available resources highlights just how influential the environment can be on the diversity present within the archaeological record. This has implications for behavioural inference, as archaeologists often associate shifts in assemblage composition with changes in behaviour.

Placing the record from the Djouroutou field site within the broader context of chimpanzee nut cracking behaviour allows us to gain insights into the disconnects between how behaviours produce material patterning. If we draw archaeological expectations from ethological observations of chimpanzees, then one might expect the resulting record to be composed of hammerstones and anvils. However, the results of our study show that the presence of such implements within a material assemblage is largely dependent on the interacting dynamics between raw material properties, tool reuse and tool transport with ecological factors including nut species and their distribution. These results have important implications for the field of Plio-Pleistocene archaeology, as stone tool variation is often interpreted as the result of technical skills [11], varying land-use strategies [88] and social transmission of information [89].

For example, the number of artefacts recovered from a site is often interpreted as the intensity of behaviour at the site or the number of individuals involved in its formation [90,91]. However, this is not the case in Djouroutou. In areas where *Coula* nuts are abundant, their soft shells [24] allow them to be cracked by a wide range of stone and wooden hammers made of different materials [60]. Therefore, the widespread accessibility of suitable stones enables multiple individuals to crack nuts simultaneously. In contrast, the scarcity of suitable stones in the northern region of Djouroutou, coupled with the fact that *Panda* nut cracking often requires large hammerstones weighing over 2 kg suggests that there are fewer stones suitable for this type of nut cracking. Consequently, the number of individuals capable of engaging in *Panda* nut cracking at any given time is likely to be restricted. Interestingly, however, the interaction of this behaviour with variation in stone material hardness across Djouroutou produces a pattern opposite to what archaeologists would expect. The areas that facilitate multi-individual nut cracking comprise the smallest lithic assemblages. In contrast, the region where nut cracking is more likely to be solitary leaves a greater number of material traces.

Given the gross differences in tool technologies, cognition and anatomy, chimpanzees cannot serve as stand-ins for hominins but serve as a useful frame reference from which to investigate aspects of hominin behaviour and biology. These results, thus, shed light on the dynamics responsible for creating material records. The variation present within the stone assemblages from Djouroutou highlights how a single percussive action can produce a diverse material signature through its interaction with the broader landscape. A result that is echoed at other primate archaeological sites [66]. Our results further this finding by highlighting how stone tool-using behaviours can be strongly conditioned by their socio-ecological context and that the resulting patterns may be highly diverse and may not intuitively reflect the behaviours that produce them.

As we continue to search for evidence of earlier tool use in the hominin lineage, the results of this study provide new insights into the material signature of a purely percussive stone tool industry. Our findings are particularly useful for periods deep within the hominin lineage when our ancestors were still living in forested environments. Although our study highlights the diversity associated with this type of tool use, these results will help to refine our search lens associated with pre-core and flake technology. This work has shown that while hammerstones and anvils are responsible for the creation of the archaeological assemblages, the resulting record is far more diverse depending on the configuration of resources. As a result, we cannot simply expect to identify a record of tool use pre-dating core and flake technology by simply searching for hammerstones. In some cases, there may be contexts where the most ubiquitous percussive tools are rare, or even completely absent, despite the presence of a widespread record of percussive activities. The earliest archaeological record may even be represented by a collection of battered fragments. Therefore, we should aim to describe the entirety of primate assemblages in the capacity we have studied hammerstones. This will help to better calibrate our search lens to the diversity of forms this early archaeological record might take on.

## 8. Conclusion

Although there is a growing consensus that tool use extends beyond its current earliest manifestation in the archaeological record, there is also a need to validate such a consensus with physical evidence. Such an endeavour requires a robust understanding of what an archaeological record, produced solely by stone percussive behaviours, may look like. Although there is a robust understanding of the identifying attributes of percussive implements such as hammerstones and anvils, there remains little understanding of how the sole use of such implements creates an archaeological record and the diversity associated with it under different environmental circumstances. Having such explicit expectations of such a record will dramatically improve our chances of detecting a pre-core and flake technology in archaeological contexts from our deep past.

The chimpanzees of Djouroutou make a substantial contribution to this topic as they use stone solely for food processing activities. When this material patterning is considered in combination with the spatial arrangement of nut trees and the various raw materials, it becomes possible to discuss the formation processes associated with a purely percussive archaeological record. Such insights are important as they allow us to identify the environmental factors that are most influential on the material manifestation of nut cracking. In doing so, this work allows us to understand the breadth of material diversity such behaviours can produce. The resultant material landscape provides insight into what a purely percussive archaeological record looks like. Thus, the relationship between chimpanzee tool behaviours, the broader environment and their material culture elucidated by this work can be generalized to discuss the earliest, yet-to-be-discovered archaeological record.

## Data Availability

Supplementary material is available online at Zenodo [92]. Supplementary material is availabe online [93].
